# Painless Operations by Alveolar Injections

**Published:** 1910-09-15

**Authors:** B. H. Masselink


### PAINLESS OPERATIONS BY ALVEOLAR INJECTIONS.

BY B. H. MASSELINK, D. D. S., L. D. S.

(Abstract from article in August *Comos.*)

I invite you to the consideration of the following simple and inexpensive method. This method is not entirely in its experimental stage. In England, where it found its impetus, it is known as "Otte's intra-osseous injection," and scores of dental surgeons are using it costanntly. In the maxilla of the posterior, middle, and anterior dental nerves form a plexus in a line drawn along the apices of the roots; that the teeth are supplied from this plexus as well as from the main nerves. In the mandible the teeth are supplied by the inferior dental, as here the supply is more direct from the main nerve.

The alveolar process consists of an outer and inner plate, with spongy bone or diploe intervening. The nerves with their branches ramify through this loose structure *en route* to the apices of the teeth. If a local anesthetic can be brought into contact with the nerves before they enter the apex of the tooth, such a nerve will be anesthetized and the tooth, so to speak, desensitized.

The operation for accomplishing the desired end is so simple and so entirely devoid of all subsequent dangers to tooth or patient that many interested readers will perhaps hesitate before attempting it. It need occupy only three or four minutes, and all may be done entirely without pain, as follows:

A pledget of cotton soaked in a saturated solution of cocain and adrenalin is applied to the mucous membrane in the depression between the roots of the teeth to be operated upon, and as near the apices as convenient. In a few moments the membrane will be sufficiently cocainized to insure the insertion of a hypodermic needle without pain.

A drop of local anesthetic is now injected beneath the mucous membrane. This completely anesthetizes the region in an instant. A No. $\frac{1}{2}$ round bur is now employed in drilling a hole through the outer plate of the alveolus. The sense of touch will guide one in this, for when the bur has penetrated the plate it will suddenly ease itself. An ordinary hypodermic syringe is now employed in injecting a local anesthetic into the diploe. This, in short, is the whole operation. It is absolutely necessary that it should be performed as aseptically as is practical, and for this reason the following precautions are taken; A small cotton roll is placed high up under the lip where this is possible. The region is bathed with a weak solution of mercury bichlorid and carefully dried. The bur used should be sterile, and for this reason is always kept in an antiseptic solution, and before being used is dipped in pure carbolic acid and then in pure vaseline, the former to render it aseptic after handling, the latter to facilitate the penetration of the soft membrane.

An extra syringe for injecting into the diploe other than the ordinary one is necessary. The needle-point of this syringe should be cut off to within about a sixteenth of an inch from the shank and left dull. This syringe is likewise kept in an antiseptic solution. The short needle will sink into the hole up to the shank, the latter blocking the way to prevent the return of the solution.

For convenience and quick work a large glass jar about four inches deep with a large cork stopper is used. Two large holes are made through the stopper for the two syringes, and several smaller ones for the burs. The jar is filled with a ten to fifteen per cent. solution of formalin to which a handful of borax has been added, the latter preventing the rusting of needles and burs.

In drilling into the alveolus it is not necessary to be on a level with the apices.

No hole should be drilled between the centrals, that spot being the line of union of the jaw. The roots of the central and lateral in the mandible are often too close together to insure safe drilling. In such a case the aperture should be made between the lateral and the canine, the directions of the roots indicating the best place for operating. Often two or three teeth can be desensitized with one injection. In the molar region the operation is more difficult at first, but after the operator has gained some experience these teeth also fall easy victims. The upper molars respond quickly if one operates on the lingual side, with the right angle—so quickly, indeed, that one is awestruck at times, wondering as to its reality. This is particularly true of the third molar; the lower molars, especially the second and third, are hard to master, but an excellent way will suggest itself in time.

The solution employed must necessarily be of the least toxic variety. The diploe is very vascular, and absorption takes place immediately. Preparations containing cocain must be avoided. The author has tried several varieties of local anesthetics, all with equal effect upon the tooth but with unequal effect upon the patient. A solution prepared according to the formula of Guido Fischer and used as directed is perfectly safe and absolutely certain.

| | |
|---|---|
| Take—Novocain | 1.000 |
| Thymol | 0 033 |
| Sodium chlorid | 0.450 |
| Distilled Water | 50.000 |

This solution should be put up at boiling temperature, and kept in a dark-colored bottle with a rubber stopper. Although normally stable it should be heated to boiling-point at least once a week. Heating does not affect the anesthetic properties of the solution. A small quantity sufficient for the day's use should be poured out into a small bottle, and to every fifteen drops one drop of adrenalin chlorid added. This preparation is now unstable and good only for that day.

A small quantity of the above solution carefully injected will produce local anesthesia lasting from fifteen to thirty minutes, without any noticeable toxic effects. It can be used in cases where cocain solutions in very small quantities would produce marked poisoning, with very little or no evil ensuing. I venture to say that nothing else should be used, unless it were a preparation less toxic even than the novocain.

The method is of great value in all cases where pain is a barrier to good work. In the preparation of cavities its merits need not be commented upon; thorough preparation becomes as simple as it is expedient. The preparation of vital teeth as abutments for bridges, where indicated, is "a joy."

In extirpation of pulps it has a special advantage through the action of adrenalin on the blood supply to the teeth. The bleeding is usually slight and easily controlled, affording excellent opportunities for immediate root-canal filling. The adrenalin chlorid constricts the vessels, thereby diminishing the blood supply to the teeth.

*What about the after-effects*

With the novocain solution no sloughing has ever been noticed. The small wound inflicted upon the gums heals in a day or two, with slight or no inconvenience to the patient. A few patients reported a slight pain a few hours after the operation. This subsequently passed off with no evil results, and its cause could not be ascertained.

The effects upon the pulp are, I believe, even less than under ordinary operations, for the tooth being temporarily deprived of a part of its blood supply—which it regains by degrees upon the absorption of the anesthetic—is free from the subsequent hyperemia that often follows the rapid excavating of cavities by burs.

As stated before, so far no toxic effects have been noticed from the use of novocain. In suspected idiosyncratic cases I should advise the dilution of the preparation by adding distilled water, or better, a normal salt solution.

I may here cite a few effects that are positively good, and first in importance is that upon the patient. The dread of having dental work done is abolished, and the patient returns with as little thought of pain as if he or she went to consult a physician. Instead of facing a succession of wailings each day, you often face a procession of children following a delighted mother, all to be examined and, of course, treated. Words fail in picturing the joy that patients express in various ways.

On the operator the effect varies with each individual. As for me, "Whereas formerly I loathed the preparation of cavities, now I love it." Instead of worrying about the sensitive cervical cavities, I now prefer them because of their easy access. To use the words of an English writer, "It is the nearest approach to Heaven that a dentist gets"—temporarily, I trust he means!

*Cases.* I will refer to a few of the scores of cases worthy of special notice.

A man of forty-five or fifty years of age, a member of Parliament, came for an examination. Among other things a gold filling at the cervical margin of the lower left canine was found defective. The patient said that when this tooth was filled seven years ago the pain incident to the operation was so intense that he swore he would have it extracted if it should ever need further attention. After explaining the reason why this tooth should be saved, I asked for permission to give it a trial. Without telling the patient what I intended doing, the whole operation was gone through, the intraalveolar injection being made between the canine and lateral. Within twenty minutes the cavity had been prepared for an inlay, and incidentally, a distal cavity in the lateral and a mesial cavity in the central. His astonishment and appreciation were expressed in words worthy of his statesmanship.

A little girl of eleven presented with a dozen filling of various materials, all of which after a year's service had given out.

The teeth were very sensitive, and this evidently accounted in part for the defective work. It was not until many repeated assurances were given that I would cause her no pain that the patient was coaxed into the much-dreaded dental chair. The sound of the engine caused her to shiver. Two cavities were prepared with no discomfort outside the buzz of the bur; later all the work was finished. A lost patient reclaimed!

A young lady who had been told that her extreme nervousness placed her beyond the scope of dental work called upon recommendation of her sister, who had experienced the "new delight." A pulp was extirpated in an upper second molar and several cavities prepared without a sound or stir.

To sum up: This method is quick. After the initial trials and possible failures, it need take only three or four minutes. No extra charge need be made on this account.

It is easy. You need only to try it to be convinced.

It is cheap—the cost is *nil.*

It is practically universal, the lower molars only presenting difficulties, more particularly the second and third.

Lastly, it is safe—as experience has proved, and I believe will never disprove, for we are going to improve.

### USEFUL HINTS.

Apply the pledget of cotton, soaked in cocain solution, immediately, and let the lip drop over it, and it will stay in place, doing its part while you arrange the necessary instruments.

Locate the roots carefully, so as not to injure the pericemental membrane.

Do not use the same needle too often. It is apt to rust slightly and weaken at the junction of the point with the shank. It may then break and lodge in the aperture. The manufacturers should make a silver needle for this purpose, to insure against this accident.

Obtain a good contact of the shank of the needle with the opening into the alveolus before the injection, so as to prevent waste of the solution and to enable you to better gage the amount injected. Keep the needle in position a moment after injection to prevent return of the fluid.

Touch the mucous membrane at the place of perforation with a little pure carbolic acid. This will largely prevent subsequent pain in the neighb‹ rhood.

Explore the cavity before using the engine, to make sure that the tooth is desensitized.

If you don't intend to fill permanently at the same sitting, cover the dentin with a thin layer of cement before inserting the temporary stopping. This insures against reinfection and makes the subsequent drying of the cavity with alcohol and hot air a painless procedure.

